# Injuries Among Pediatric Age Group Children Attending the Emergency Department of Maternity and Children Hospital, Buraidah City

**DOI:** 10.7759/cureus.77545

**Published:** 2025-01-16

**Authors:** Ali M Alshardan, Chandra Sekhar

**Affiliations:** 1 Family Medicine, Family Medicine Academy, Qassim Health Cluster, Buraidah, SAU

**Keywords:** buraidah city, mch hospital, pediatric injuries, place of injury, severity of trauma, time of injury

## Abstract

Background

Pediatric age group injuries is one of the burdens on a nation’s GDP and parents’ income and productivity. Multiple factors cause such injuries, and some of them can be prevented by simple care from the parents and their knowledge and practice at the time of injury. This study aims to determine the prevalence of injuries and associations between demographic factors and other risk factors with the type of injuries and severity of injuries.

Methodology

A cross-sectional study was conducted among 391 parents of the pediatric age group children (0-14 years) at the Maternity and Children Hospital’s emergency department using a validated questionnaire, a combination of interviews, and a self-administered questionnaire. Informed consent was obtained from each parent participant. Data were entered and analyzed using SPSS software version 21.0 (IBM Corp., Armonk, NY, USA).

Results

The average age of injured children was five years with a standard deviation of 3.13 years, and 64.5% (n = 252) were males. About 76.5% (n = 299) of injuries occurred at home and 14.1% (n=55) required hospital admission. Around 91.3% (n = 357) of children recovered, while 8.4% (n = 33) received alternative care. Approximately 81.3% (n = 318) of parents were unaware of basic life support (BLS). Among the children, 9.4% (n = 25) of those under five years had burns compared to 3.2% (n = 4) of those over five years, with a statistically significant association between age and burns (p < 0.05).

Conclusions

Based on the study findings, three-fourths of pediatric injuries occurred at home, of which more than 90% of injuries recovered. Still, in one-tenth of injuries, parents opted for alternative care, and more than 80% were unaware of BLS. This study recommends that health administrators and policymakers create awareness regarding BLS training for the general population along with the importance of alternative care in injuries.

## Introduction

Significant morbidity and mortality are associated with trauma among the pediatric age group children and represent one of the public health problems worldwide in both developed and developing countries [1,2]. Trauma can be classified based on its etiology as either blunt or penetrating trauma [3]. Traumatic injuries have long-lasting impacts on all aspects of victims’ lives, causing discomfort and disabilities associated with the injuries.

According to a World Health Organization (WHO) report, about 2,000 children under the age of 14 years die every day globally [4]. Pediatric injuries can have long-term health, educational, and social consequences in later life, such as physical disability, psychological morbidity, cognitive and social impairment, and lower educational achievement [5]. Traumatic injuries from all causes result in 9% mortality in children and appear to be on the rise in the Arab Gulf States, similar to higher-income countries such as the United States [6]. Pediatric burn injuries, in particular, are a major concern, as burns are among the most common causes of hospitalization in children under five years, with scald burns being predominant due to domestic hazards such as hot liquids and steam [7].

Saudi Arabia is no exception for various childhood injuries which have been increasing drastically over some time. The fatality rate associated with motor vehicle crashes in Saudi Arabia is estimated to be double that of the United States [8]. Studies conducted in Saudi Arabia have highlighted the prevalence of campfire burns, especially in crawling infants, emphasizing the need for better preventive strategies and parental awareness regarding thermal injuries [9]. Several studies have been conducted in the capital of Saudi Arabia. One study conducted at King Abdulaziz Medical City showed that scald burn injury was the most common in children aged one to three years. Burn injuries can occur due to domestic activities such as cooking, electricity, exposure to chemicals, and fireworks [10,11].

However, in the Qassim region, which has a population of about 1.6 million, of which about 24% are younger than 14 years [12], little is known about the epidemiology and patterns of injury, such as the severity or mechanism of injury. Injuries occur because of several factors, including economic status, age, and education [13]. In addition, based on a Google Scholar search, the literature in Qassim Province was scarce regarding beliefs and parental and public awareness levels concerning childhood injury prevention and when to seek medical advice.

A single-center study conducted in the Qassim region reported that the most frequent trauma among children was road traffic accidents at 52% and falls from height at about 30%, with high mortality observed in comparison to international benchmarks [14]. Another study conducted in 2020 in Qassim Province reported that 46.3% of home-related injuries were observed in the youngest child [15]. These findings align with regional studies in Oman, which emphasized the need for trauma systems that address the unique demographic and cultural challenges of the Middle East [6]. It is essential to consider a child’s age when developing injury prevention strategies, as growing and developing children have different needs. It is also important to understand the etiology and spectrum of trauma in children to develop effective prevention strategies.

A study conducted in the Riyadh Emergency Department on pediatric injuries found that two-thirds of the patients were males. The most common injuries were fractures, dislocations, and subluxations, followed by penetration injuries and burns. Falls, hot liquids, and chemical exposure were the leading causes. The study concluded that these mostly preventable non-traffic injuries were significantly associated with pediatric mortality and morbidity [16].

A study at the Suez Canal University Hospital in Egypt examined musculoskeletal injuries in 270 children. In this study, falls caused 66% of injuries, with 44% occurring at home, 30% on roads, 17% at playgrounds, and 9% in schools. Around 80% of injuries involved the upper limbs. The study found that injury prevalence and characteristics varied by age group, likely due to behavioral changes with age [17].

A study in South India (2015-2017) by Prakash Raju et al. focused on children aged under 12 years at an emergency department of a tertiary care hospital. The study found that 63.9% of injuries occurred at home, mainly due to falls. Nearly 40% of road traffic accident injuries involved two-wheelers, and 59% of children had head and maxillofacial injuries. Approximately 72.6% of children experienced mild trauma based on the Pediatric Trauma Score [18].

This study aims to close the knowledge gap and develop an appropriate approach (health promotion measures to parents) toward injury prevention in children based on the knowledge of the epidemiology of trauma considering the high diversity of injuries. The study aims to determine the prevalence of injuries, associated factors, and their association with the type of injuries.

## Materials and methods

Study setting and design

The study was conducted at Maternity and Children’s Hospital (MCH), which has 300 beds, including 40 beds for the emergency department. MCH is a referral center for pediatrics in the Al-Qassim region, located in Buraidah, the region’s capital. This study’s cross-sectional design was selected because the study design can analyze data collected from a group of participants at one point in time.

Sample size and sampling

Based on a previous study that reported a 52% prevalence of injury among the pediatric age group [4], in this study, we considered the prevalence of injury at 52%, with an error of 5%, and a confidence interval of 95%. We applied all three above parameters in the WHO statistical software for sample size calculation. The sample size was calculated to be 384. However, we collected data from 391 participants to maintain accuracy in interpreting the results. A non-probability convenience sampling technique was used. Any patient who met the inclusion criteria and was available during data collection was invited to complete the questionnaires voluntarily.

Inclusion and exclusion criteria

All pediatric patients visiting the MCH emergency department for trauma (0-14 years of age, based on the definition from the Ministry of Health) who agreed to participate in the study were included. Exclusion criteria included trauma due to medical conditions, such as falls due to seizure disorder, and birth trauma.

Questionnaire development

A formulated validated questionnaire was used for data collection. The questions were designed based on the previously published literature [14,15] and the objectives of this study. We conducted a pilot study before validating to assess understanding of the questionnaire, followed by translation and back-translation to ensure accuracy. The data collection tools consisted of two sections. The first section of the questionnaire included demographic information, such as age, gender, education level, family income, and mother’s occupation. The second section comprised information related to the trauma, such as the site of trauma, type of trauma, and outcome of the trauma. Data collection was performed between June 2024 and August 2024. The principal investigator used a combination of interviews and a self-administered questionnaire to collect the data from participants.

Statistical analysis

The data gathered were entered into SPSS version 21.0 (IBM Corp., Armonk, NY, USA) for data analysis. The quantitative variables, such as age, were presented as mean ± standard deviation (SD). Categorical data such as gender, year of the accident, and age categories were presented as frequencies and percentages. A test with a p-value <0.05 was considered statistically significant. The dependent variables in our study were the type of injury and severity of the trauma. The chi-square test was applied to determine the association between the type of injury and severity of trauma with demographic and other risk factors such as the occupation of the parent and the working hours of the mother. An independent t-test was employed for the number of children in the family versus the severity of trauma.

Ethical considerations

The research proposal was submitted to the regional ethics committee for approval (approval number: 607-45-12633; dated March 14, 2024). After obtaining approval from the Institutional Review Board of Qassim Health Cluster, the proposal letter was sent to the MCH director for information and facilitation of the research. All respondents provided informed written consent, and confidentiality of their information was maintained. Data were not shared with any public or private agencies.

## Results

In this study, 391 children participated. We distributed our questionnaire to the parents of children through the emergency department of MCH in Qassim province. The response rate in the study population was 87% (391/450).

The mean age of the children included in the study was 5.0 ± 3.13 years. Similarly, the mean average basic life support (BLS) received status of parents was 3.73 ± 4.32 years. In the study population, 91.3% (n = 380) of parents were married, and 4.3% (n = 17) were widowed and divorced. Regarding fathers’ educational level, about 9.2% (n = 36) had completed primary education, 28.9% (n = 113) had completed secondary education, 49.4% (n = 194) had finished college-level education, and 12.3% (n = 48) had finished postgraduation (PG) and above education. Regarding mothers’ educational level, about 12% (n = 47) had completed primary education, 15.6% (n = 61) had completed secondary education, 64.7% (n = 253) had acquired a college education, and only 7.7% (n = 30) had completed PG and above education.

Regarding parental income, 9.5% (n = 37) earned ≤5,000 SR/month, 28.4% (n = 111) earned between 5,001 and 10,000 SR/month, and 62.1% (n = 243) earned >10,000 SR income/month. In this study, about 64.5% (n = 252) were males. Regarding the occupation of children’s mothers, about 50.6% (n = 198) were housewives, 42.7% (n = 167) had a government job, and 6.6% (n = 26) had a private job. Nearly 46% (n = 180) of mothers worked eight hours/day. About 5.1% (n = 20) were working 9-12 hours/day, and only 2.6% (n = 10) were working more than 12 hours/day (Table 1).

**Table 1:** Demographic characteristics of the study participants.

Demographic variables	Number of participants (n)	Percentage (%)
Age ± SD	5.0 ± 3.13 years	
≤5 years	266	68.0
>5 years	125	32.0
Nationality		
Saudi	380	97.2
Non-Saudi	11	2.8
Gender		
Male children	252	64.5
Female children	139	35.5
Occupation of the mother		
Govt. job	167	42.7
Private job	26	6.6
Housewife	198	50.6
Mother hours of work/day		
8 hours	180	46.0
9–12 hours	20	5.1
>12 hours	10	2.6
Not applicable (housewives)	181	46.3

In this study, approximately 76.5% (n = 299) of injuries occurred at home, 8.7% (n = 34) at playgrounds, 8.2% (n = 32) at roads, and, lastly, 6.6% (n = 26) at school. Concerning the time of injury, about 61.9% (n = 242) of injuries occurred in the evening/night. Approximately 86.4% (n = 338) reached the healthcare center within two hours. Nearly 95.4% (n = 373) of participants did not utilize an ambulance facility. Regarding the type of injuries in the study group, 59.6% (n = 233) had injuries due to falls (without any external force), 25.6% (n = 100) due to trauma, 7.4% (n = 29) due to burns, and 1.3% (n = 5) due to road traffic accidents (Table 2).

**Table 2:** Injury characteristics (place, time, and type) status among the study participants.

Injury-related variables	Number (n)	Percentage (%)
Place of injury		
Home	299	76.5
Road	32	8.2
School	26	6.6
Playground	34	8.7
Time of injury		
Morning	64	16.4
Afternoon	85	21.7
Evening/Night	242	61.9
Time to reach the health center		
Within 2 hours or less	338	86.4
3–4 hours	22	5.6
5–6 hours	8	2.0
>6 hours	23	5.9
Ambulance utilized		
Yes	18	4.6
No	373	95.4
Type of injury		
Fall (without any external force)	233	59.6
Road traffic accident	5	1.3
Drowning	4	1.0
Burns	29	7.4
Trauma	100	25.6
Others	12	3.1
Ingestion of chemical	8	2.0

In this study, only 14.1% (n = 55) of injuries required admission. Moreover, 91.3% (n = 357) of children recovered from the injuries. Further, 8.4% (n = 33) of children received alternative care. Concerning BLS awareness of parents, about 81.3% (n = 318) were not aware (Table 3). Among the alternative care users, about 30.3% (n = 10) applied bandages, 15.1% (n = 5) applied cold compression, and 12.1% (n = 4) applied cloves. Approximately 9.09% (n = 3) of participants received alternative care of applied turmeric, flour, and Vicks (Table 4).

**Table 3:** Severity, outcome of injury, alternative care, and mothers’ BLS awareness status in the study population. BLS: basic life support

Variables	Number (n)	Percentage (%)
Severity of trauma		
Required admission	55	14.1
Not required admission	336	85.9
Outcome of injury		
Died	2	0.5
Recovered	357	91.3
Partial recovery	29	7.4
Status not known	3	0.8
Received alternative care		
Yes	33	8.4
No	358	91.6
Parents’ BLS awareness status		
Yes	73	18.7
No	318	81.3

**Table 4:** Type of alternative care received in the study population.

Type of alternative care	Number (n)	Percentage (%)
Apply bandage	10	30.3
Apply cold compression	5	15.1
Apply clove	4	12.1
Apply flour	3	9.09
Apply turmeric	3	9.09
Apply Vicks	3	9.09
Apply coffee powder	1	3.03
Apply mouthwash	1	3.03
Apply myrrh	1	3.03
Apply tomato sauce	1	3.03
Olive oil with salt	1	3.03
Total	33	100

On applying the chi-square test to compare the categories of mothers’ working hours, time of injury, and living status of the children with the type of injury, no significant association was observed between these categories (p > 0.05). This analysis is not presented in the tables.

In the 0-5-year age group children, 13.5% (n = 36) required admission, whereas in the 6-13-year age group, 15.2% (n = 19) required admission. In the male gender, the required admission prevalence was 11.9% (n = 30), and in the female gender, the required admission prevalence was 18% (n = 25). Similarly, in those with <5,000 SR/month income, 18.9% (n = 25) required admission. Among those earning 5,001-10,000 SR/month, the admission requirement was 13.5% (n = 15). Finally, among those earning >10,000 SR/month, about 13.6% (n = 33) of children required admission for their injuries. The statistical analysis showed no statistically significant association between the severity of trauma in relation to hospital admission versus age category, gender, and monthly family income (p > 0.05) (Table 5).

**Table 5:** Demographic factors associations with the severity of trauma in the study population. SR: Saudi riyal

Demographic factors	Required admission	Not required admission	Total	Chi-square value	P-value
Age category					
0–5 years	36 (13.5%)	230 (86.5%)	266 (100.0%)	0.195	0.659
6–13 years	19 (15.2%)	106 (84.8%)	125 (100.0%)		
Gender					
Male	30 (11.9%)	222 (88.1%)	252 (100.0%)	2.740	0.098
Female	25 (18.0%)	114 (82.0%)	139 (100.0%)		
Family income/month					
<5,000 SR	7 (18.9%)	30 (81.1%)	37 (100.0%)	0.796	0.672
5,001–10,000 SR	15 (13.5%)	96 (86.5%)	111 (100.0%)		
>10,000 SR	33 (13.6%)	210 (86.4%)	243 (100.0%)		

In the study population, among the <5-year age group, 9.4% (n=25) of children had burns, 62.8% (n = 167) suffered from falls, 19.5% (n = 52) experienced trauma, and 1.5% (n = 4) drowned. In the >5-year age group, 3.2% (n = 4) had burn injuries, 52.8% (n = 66) had fall injuries, 38.4% (n = 48), and none drowned. The association between the type of injuries, such as the prevalence of burns, and the <5-year age group of children was statistically significant (p < 0.05). Similarly, a high prevalence of trauma at 29% (n = 73), falls at 58.7% (n = 148), burns at 7.5% (n = 19), and drowning at 0.4% (n = 1) was observed in the male gender. Regarding injuries among females, about 19.4% (n = 27) were from trauma, 61.2% (n = 85) were from falls, 7.2% (n = 10) were from burn injuries, and 2.2% (n = 3) were from drowning. There was a statistically significant association between the male gender and trauma prevalence (p < 0.05). Overall, 18.9% (n = 7) of injuries were from trauma in those with an income <5,000 SR/month, 23.4% (n = 26) in those with an income of 5,001-10,000 SR/month, and 27.6% (n = 67) in those with an income >10,000 SR/month group. Monthly family income and different types of injuries were not statistically significant (p > 0.05) (Table 6).

**Table 6:** Sociodemographic factors associated with the type of injury in the study population. *: significant. RTA: road traffic accident; SR: Saudi riyal

Variables	Fall	RTA	Drowning	Burns	Trauma	Others	Ingestion of chemical	Chi-square value	P-value
≤5 years	167 (62.8%)	1 (0.4%)	4 (1.5%)	25 (9.4%)	52 (19.5%)	10 (3.8%)	7 (2.6%)	27.51	0.001*
>5 years	66 (52.8%)	4 (3.2%)	0 (0.0%)	4 (3.2%)	48 (38.4%)	2 (1.6%)	1 (0.8%)		
Male	148 (58.7%)	3 (1.2%)	1 (0.4%)	19 (7.5%)	73 (29.0%)	7 (2.8%)	1 (0.4%)	15.67	0.016*
Female	85 (61.2%)	2 (1.4%)	3 (2.2%)	10 (7.2%)	27 (19.4%)	5 (3.6%)	7 (5.0%)		
≤5,000 SR	27 (73.0%)	0 (0.0%)	0 (0.0%)	1 (2.7%)	7 (18.9%)	1 (2.7%)	1 (2.7%)	9.77	0.635
5,001–10,000 SR	69 (62.2%)	1 (0.9%)	0 (0.0%)	8 (7.2%)	26 (23.4%)	3 (2.7%)	4 (3.6%)		
>10,000 SR	137 (56.4%)	4 (1.6%)	4 (1.6%)	20 (8.2%)	67 (27.6%)	8 (3.3%)	3 (1.2%)		

## Discussion

Pediatric injuries continue to be a significant global health concern, leading to considerable morbidity and mortality. Our study mirrors global trends while highlighting specific characteristics unique to our region. Injuries predominantly affected male children (64.5%) with an average age of five years, reflecting their tendency to engage in riskier activities, a trend consistently noted in the literature [6,19]. Most injuries occurred at home (76.5%), underscoring the urgent need to enhance safety measures within domestic settings. Similar patterns have been observed in studies from Saudi Arabia and the United Arab Emirates, where household falls and burns constitute a large proportion of pediatric injuries [10,14,20].

In our study, falls emerged as the leading cause of injuries (59.6%), followed by trauma (25.6%) and burns (7.4%). These findings are consistent with other regional studies. In Riyadh, falls were the most common cause of injury among children, accounting for 57.8% of cases, with burns at 9.9%, and blunt trauma (including road traffic accidents) at 19.1% [16]. Similarly, in Makkah, Gari et al. (2012) found that burns constituted 34% of pediatric injuries, highlighting a significant prevalence of burn injuries in that region [21]. In the Eastern Mediterranean region, Othman and Kendrick (2010) reported that burns were responsible for 17% of pediatric injuries, while falls accounted for 20-50% across different countries [22]. The differences in the prevalence of injuries could be multifactorial.

Importantly, our study found a statistically significant difference in the prevalence of burns among children under five years old (9.4%) compared to those over five years old (3.2%) (p < 0.05). Scald burns were more frequently observed among children under five years old, often attributed to hot beverages and water, a pattern noted across various studies in Saudi Arabia (72% of burn cases) [10]. Similarly, a study in Iran found that 69% of pediatric burns were scalding in children under five, reinforcing the need for preventive measures targeting this vulnerable group [23]. This highlights the cultural and environmental factors that influence injury mechanisms and calls for customized prevention strategies to address these specific risks.

Gender differences in trauma prevalence were also statistically significant, with males experiencing a higher rate of injuries (29%) compared to females (19.4%) (p < 0.05). Similar trends have been observed in both local and international studies, which often attribute this disparity to differences in activity levels and risk-taking behaviors between boys and girls [19,24]. While this aligns with the global understanding of gender differences in injury epidemiology, it highlights the need for targeted, gender-sensitive interventions.

Our study found that most injuries occurred during periods of increased activity and potentially reduced supervision. While specific percentages vary, it is widely recognized that a significant proportion of pediatric injuries occur during afternoons and evenings when children are more active and supervision may be less vigilant [25]. This finding reinforces the importance of parental education and supervision strategies during these high-risk periods.

Despite existing challenges in prehospital care, only 4.6% of injured children in our study utilized ambulance services. While we observed a high recovery rate (91.3%) and a low admission rate (14.1%), suggesting that many injuries were minor, concerns arise when comparing these findings to data from tertiary centers in Riyadh and Jeddah. These centers report higher intensive care unit (ICU) admissions and mortality rates for severe cases, pointing to gaps in resources and trauma system preparedness [12,26]. A study documented an ICU admission rate of 12% and a mortality rate of 5% among pediatric trauma patients [8]. These findings suggest that while outcomes for minor injuries are generally favorable, there is a pressing need to improve trauma care for severe injuries.

Burn injuries, though less frequent, were notably concentrated among younger children, with a prevalence of burns of 9.4% in those <5 years of age, with scald burns being the most common type in our study. Studies from Riyadh reported 68% of burns in under five-year-old children [10]. Additionally, another study from the Asir region of Saudi Arabia reported that 74% of pediatric burn patients were under five years old, further emphasizing the need for focused preventive strategies [27]. Similar trends were reported in Turkey, where younger children were more vulnerable to burns due to limited awareness and unsafe domestic practices [22]. Interestingly, while most burns in our study were minor, tertiary care studies report higher rates of hospitalization and ICU admission for burns exceeding 10% of the total body surface area (TBSA), indicating a need for standardized thresholds in burn care management [16,21].

A striking gap in parental knowledge was observed, with 81.3% of parents lacking BLS training. This finding aligns with results from studies in Saudi Arabia and Egypt (87% untrained parents), which consistently highlight low levels of first-aid awareness among parents [17]. Interventions that improve parental knowledge have demonstrated significant impact, as demonstrated by a campaign on shaken baby syndrome in the Eastern Province of Saudi Arabia, which significantly enhanced parental understanding of injury prevention [18,28].

When compared to international benchmarks, differences in pediatric injury outcomes are evident, which could be due to hospital settings, age groups of the children, parental occupation, and literacy. The children in Saudi Arabia exhibit higher mortality rates for severe injuries compared to their counterparts in the United States, largely due to differences in prehospital care and trauma system maturity [8,24]. The U.S. National Trauma Data Bank serves as a gold standard for benchmarking trauma outcomes and could be instrumental in guiding similar initiatives in Saudi Arabia in all provinces, as well as in other Middle Eastern countries [29].

Our study, with its large sample size and focus on pediatric injuries in the Qassim region, provides valuable insights into injury patterns and demographics. It addresses a critical gap in local data, aiding in developing prevention strategies. However, reliance on parental reporting may introduce recall bias, and the lack of long-term follow-up data limits our understanding of the lasting impact of these injuries. Future research should include longitudinal studies to monitor recovery and evaluate interventions for vulnerable groups, such as younger children and those from low-income backgrounds, to enhance injury prevention and management.

## Conclusions

This study highlights critical gaps in pediatric injury prevention and management while providing actionable insights based on regional and international findings. Collaborative efforts among healthcare providers, policymakers, and communities are essential to implement effective interventions, ultimately reducing the burden of preventable injuries and improving health outcomes for children globally. This study offers practical recommendations. Enhancing parental education with targeted BLS and first-aid training campaigns is crucial, especially in areas with low prehospital care use. Improving safety regulations to address domestic hazards such as hot water scalds and unsecured furniture is also vital. Strengthening trauma systems, including prehospital care and specialized trauma units, and implementing age-specific and gender-sensitive prevention programs can reduce risks and improve outcomes. Establishing trauma registries modeled after the U.S. National Trauma Data Bank would support data-driven improvements in trauma care.

## Appendices

Study questionnaire

Demographic characteristics

1. Age of the child: ------------(in years)

2. Gender of the child: 1. Male 2. Female

3. Nationality: 1 Saudi 2. Non-Saudi

4. Marital status of child parents: 1. Married 2. Divorced 3. Widowed

5. Living status of the children: 1. With one Parent (mother/father) 2. With both parents 3. With others (Grandparents/uncle/aunt)

6. Education of the mother: 1. Primary school 2. High school 3. College level 4. PG and above

7. Education of the father: 1. Primary school 2. High school 3. College level 4. PG and above

8. Family income/month: 1. ≤5,000 SR 2. 5,001-10,000 SR 3. >10,000 SR

9. Occupation of mother: 1. Housewife 2. Government job 3. Private job

10. Hours of work per day of mother: 1. 8 hours 2. 9-12 hours 3. >12 hours 4. Not applicable

11. Number of children in the family: --------------------

12. Place of injury: 1. Home 2. Road 3. School 4. Playground 5. Other (specify) -------

13. Time of injury: 1. Morning 2. Afternoon 3. Evening/Night

14. In case of injury, at what time reach to healthcare service/hospital: 1. Within 2 hours or less 2. 3-4 hours 3. 5-6 hours 4. >6 hours

15. Did the child utilize an ambulance? 1. Yes 2. No

16. Site of injuries: 1. Extremities 2. Abdomen. 3. Head injury 4. Chest 5. Other injuries (specify) ------------------

17. Type of injury: 1. Fall (without any external force) 2. Road traffic accident (RTA) 3. Drowning 4. Burns 5. Assaults and abuse 6. Trauma 7. Others (Specify)----------.

18. Severity of trauma: 1. Required admission 2. Not required admission

19. Outcome of injury: 1. Died 2. Recovered 3. Partial recovery 4. Status not known

20. Before arrival at the hospital, have you received any alternative care (other than the allopathy system of Medicine)? 1. Yes 2. No

21. If yes, what alternative care they received? -------------

22. Do parents know about basic life support (BLS) training? 1. Yes 2. No                                   

23. If yes, when did the parent receive BLS training? -----------
